# Individual Differences in Verbal and Spatial Stroop Tasks: Interactive Role of Handedness and Domain

**DOI:** 10.3389/fnhum.2017.00545

**Published:** 2017-11-10

**Authors:** Mariagrazia Capizzi, Ettore Ambrosini, Antonino Vallesi

**Affiliations:** ^1^Department of Neuroscience, University of Padova, Padova, Italy; ^2^San Camillo Hospital IRCCS, Venice, Italy

**Keywords:** hemispheric lateralization, brain asymmetries, spatial processing, verbal processing, hand preference

## Abstract

A longstanding debate in psychology concerns the relation between handedness and cognitive functioning. The present study aimed to contribute to this debate by comparing performance of right- and non-right-handers on verbal and spatial Stroop tasks. Previous studies have shown that non-right-handers have better inter-hemispheric interaction and greater access to right hemisphere processes. On this ground, we expected performance of right- and non-right-handers to differ on verbal and spatial Stroop tasks. Specifically, relative to right-handers, non-right-handers should have greater Stroop effect in the color-word Stroop task, for which inter-hemispheric interaction does not seem to be advantageous to performance. By contrast, non-right-handers should be better able to overcome interference in the spatial Stroop task. This is for their preferential access to the right hemisphere dealing with spatial material and their greater inter-hemispheric interaction with the left hemisphere hosting Stroop task processes. Our results confirmed these predictions, showing that handedness and the underlying brain asymmetries may be a useful variable to partly explain individual differences in executive functions.

## Introduction

“*I may be left-handed, but I’m always right!*” is just one of the many quotes that circulate on the web ironically attesting that left-handedness has been traditionally associated with negative value and connotation. As an example, consider that in some societies left-handed children were often forced to use the right hand for those tasks they would naturally perform with their left hand such as writing (e.g., Klöppel et al., 2007). Left-handedness is now more widely accepted, though right-handed people still make up the majority of the population (~90%; Corballis, 2003; see also Peters et al., 2006).

Contrary to popular belief, hand preference represents a valuable opportunity that nature provides us with to explore the hemispheric organization of the human brain. Summing up the key findings from previous neuroanatomical studies (e.g., Witelson, 1985, 1989; Habib et al., 1991; Witelson and Goldsmith, 1991; Tuncer et al., 2005), non-right-handers (i.e., left- and mixed-handers) would have on average a larger corpus callosum than right-handers. This implies better inter-hemispheric interaction, which means better coordination across both hemispheres, for left-handers compared to right-handers (e.g., Cherbuin and Brinkman, 2006). Mixed-handedness has also been associated with increased right hemispheric activity at rest (e.g., Propper et al., 2012).

What do these anatomical and functional differences between non-right- and right-handers tell us about cognitive functioning? Specifically, can handedness give enhanced insights into individual differences in behavioral performance and, if so, to what extent? This intriguing question has stimulated a great deal of work with mixed results so far. In a recent review, Prichard et al. (2013) concluded that, to overcome the current impasse on the topic, it is necessary to move away from the use of direction of hand preference, resting on the comparison of left- vs. right-handers, and focus instead on consistency of handedness, comparing inconsistent/mixed-handers (ICH) vs. consistent/strong-handers (CH). Relative to ICH, CH use “the dominant hand for virtually all common manual activities” (Prichard et al., 2013, p. 1). In line with Prichard et al. (2013), consistency of handedness has been shown to be a good predictor of performance in many cognitive domains. Specifically, ICH exhibit superior performance on tasks that require access to right-hemisphere processes and that implicate inter-hemispheric interaction, such as memory retrieval and belief updating/cognitive flexibility tasks (e.g., Jasper and Christman, 2005; Propper et al., 2005; Lyle et al., 2012). Overall, these findings have been taken as evidence for the argument that “consistent vs. inconsistent handedness is associated with decreased vs. increased interhemispheric interaction and with decreased vs. increased right hemisphere access, respectively” (Prichard et al., 2013, p. 1). However, the debate about whether hand preference, and in particular consistency of handedness alone, represents a useful variable to explain performance is far from over (e.g., Hardie and Wright, 2014). The main reasons are briefly outlined below.

The distinction between CH and ICH is usually based on the median split on scores in one of the most widely used questionnaires to measure handedness, namely, the Edinburgh Handedness Inventory (EHI; Oldfield, 1971; see also Edlin et al., 2015). When the median split is performed on the raw EHI scores, direction of handedness and consistency of lateralization may be conflated. In such a case, indeed, the consistent group is composed of consistent right-handers only, whereas the inconsistent group includes inconsistent right-handers, inconsistent left-handers and consistent left-handers. The same problem still holds for those studies that exclude consistent left-handers from the analyses (e.g., Propper et al., 2005). To avoid this, a common procedure is to perform the median split on the absolute EHI scores, instead of the raw ones, to group CH (whether left or right) into one category and ICH (whether left or right) into another category (e.g., Lyle et al., 2012). This approach too, however, might be criticized to the extent that dichotomization of a continuous variable (like the EHI scores) into a categorical measure can lead to biased results (e.g., DeCoster et al., 2009). Taking into account all these issues related to handedness as a categorical variable, here we performed polynomial regression analyses on the continuous EHI scores to explore both direction and consistency of handedness. We will elaborate further on this issue in the “Data Analysis” section.

To investigate the relationship between handedness and cognitive functions, the present study focused on the Stroop task (Stroop, 1935; MacLeod, 1991). In a typical color-word Stroop paradigm, participants are presented with words denoting different colors. The association between the ink color in which the word is displayed and the meaning of the word can be either congruent (e.g., RED printed in red) or incongruent (e.g., RED printed in blue). The participant’s task is to identify the ink color of the word and ignore its meaning. A robust finding that emerges in the Stroop task is the so-called “Stroop effect”, which refers to a drop in performance in incongruent compared to congruent color-word matching.

A general agreement exists that successful performance in the Stroop task requires the ability to overcome a prepotent and automatic tendency (i.e., reading the word) in order to implement, in its place, a less spontaneous process (i.e., identifying the ink color), a series of operations that collectively tap into the construct of *cognitive* *control* (e.g., MacDonald et al., 2000; Koechlin et al., 2003; Braver, 2012). Converging evidence from neuropsychological (e.g., Perret, 1974; Gläscher et al., 2012; Tsuchida and Fellows, 2013; Geddes et al., 2014; Cipolotti et al., 2016), structural (e.g., Vallesi et al., 2017) and functional magnetic resonance imaging (fMRI) data (e.g., Floden et al., 2011; see Derrfuss et al., 2005; Laird et al., 2005; Cieslik et al., 2015, for meta-analyses) points to the selective involvement of left brain areas in the color-word Stroop task, corroborating the claim that some executive functions may be fractionated along the left-right axis of the human brain (see Stuss, 2011; Vallesi, 2012).

Based on previous findings on handedness and Stroop (Christman, 2001), we predicted worse performance for non-right-handers compared to right-handers in a typical color-word Stroop paradigm, a task mainly lateralized to the left side of the brain. According to Christman (2001), left-handers would be impaired at keeping word and color dimensions of Stroop stimuli separate because of their greater degree of inter-hemispheric interaction. Importantly, the same study also showed that left-handers outperformed right-handers on a version of the local-global task requiring integration of left and right hemispheric processes. These findings open up the possibility that non-right-handers could hence outperform right-handers should the Stroop task involve spatial rather than verbal material. Indeed, reasoning that processing of spatial information recruits more the right hemisphere (e.g., Deutsch et al., 1988; Shulman et al., 2010), and that the cognitive processes underlying the Stroop task tend to be lateralized to the left one (e.g., Floden et al., 2011; Gläscher et al., 2012; Tsuchida and Fellows, 2013; Geddes et al., 2014; Cipolotti et al., 2016), it is conceivable to expect that greater collaboration between the two hemispheres should result in better performance in this context. Supporting our rationale, there is evidence that left-handers are facilitated in tasks that engage the right hemisphere for visuo-spatial activities (e.g., Beratis et al., 2013). For example, these authors showed that left-handers performed better than right-handers on the Trail-Making Test-B (TMT-B), a task that has been related to the functioning of the right hemisphere (e.g., Jacobson et al., 2011; Kopp et al., 2015).

In sum, to our knowledge, no study has so far directly compared verbal and spatial Stroop tasks as a function of handedness within a single experimental session. This manipulation allows exploring whether hand preference may explain individual differences in tasks that target the same cognitive operation (i.e., resistance to interference) but that diverge in the degree of inter-hemispheric interaction they require. Moreover, our protocol may partly add to the understanding of the hemispheric organization of executive functions in the brain, an issue that still remains debated and poorly understood (e.g., Badre and D’Esposito, 2007; Jacobson et al., 2011; Kim et al., 2012; Geddes et al., 2014; Babcock and Vallesi, 2015; Capizzi et al., 2016; Cipolotti et al., 2016; for a review, see Vallesi, 2012).

## Materials and Methods

### Participants

An initial sample of 246 University students took part in the study as part of a larger research project, for which they were asked to fill in the EHI among other questionnaires. This allowed us to categorize them according to their hand preference. Forty-three extra participants (41 with an EHI score below 0 and 2 with an EHI score equal to 0) were then recruited through social media advertisements targeting non-right-handers in order to have an appropriate sample belonging to this population. However, such participants were debriefed on the precise nature of the study only once the experimental session was concluded^1^.

Data from two participants were discarded due to poor performance (<50% accuracy in either of the two Stroop tasks). The remaining 287 participants (mean age: 23.3 years, age range: 19–34 years, 171 females) were included in the analyses. All participants had normal or corrected-to-normal visual acuity and reported normal color vision. Participants were compensated for their time and gave written informed consent prior to participation. The procedure of the study was approved by the Bioethical Committee of the Azienda Ospedaliera di Padova and the study was conducted according to the guidelines of the Declaration of Helsinki.

Handedness was determined with the original version of the EHI (Oldfield, 1971), which provides a score ranging from −100 (extreme left-handedness) to +100 (extreme right-handedness). In our sample, 232 participants (mean age: 23.28 years, age range: 19–34 years, 140 females) had EHI scores above 0 (mean score = 82.31, *SD* = 17.23, range = 10–100), 52 (mean age: 23.29 years, age range: 20–34 years, 30 females) below 0 (mean score = −61.15, *SD* = 31.32, range: −10 to −100) and 3 (mean age: 24.33 years, age range: 23–25 years, 1 female) had no overall preference (EHI score = 0). The EHI score mean of the whole sample was 55.45 (*SD* = 59.19) with an EHI score median of 80.

### Tasks and Procedure

Participants were tested individually in a quiet and normally illuminated room. The color-word and the spatial Stroop tasks were shortened versions of the ones used in Puccioni and Vallesi (2012a,b). The two Stroop tasks were presented in a counterbalanced order across participants along with other behavioral tasks not reported here.

In the color-word Stroop task, stimuli consisted of four Italian color words: ROSSO (red in Italian), BLU (blue), VERDE (green) and GIALLO (yellow). Each word was presented individually in one of four ink colors (red, blue, green and yellow) in such a way to yield congruent and incongruent color-word pairings. Participants were required to identify the ink color and ignore the meaning of the word through a key press on the computer keyboard.

In the spatial Stroop task, stimuli consisted of four arrows pointing to one of the four corners of the screen (i.e., upper-left, upper-right, lower-left and lower-right). Each arrow was presented individually in one of the four quadrants of the screen resulting in congruent (e.g., upper-left pointing arrow positioned in the upper-left quadrant) and incongruent conditions (e.g., upper-left pointing arrow positioned in the lower-right quadrant). Participants had to respond according to the pointing direction of the arrow and ignore the corresponding position through a key press.

For both color-word and spatial Stroop tasks, stimuli (words or arrows, respectively) were presented for 500 ms and then replaced by a 2000 ms blank response screen. The next trial appeared after an inter-trial interval that lasted randomly and continuously between 250 ms and 700 ms. Each Stroop task consisted of two blocks of 64 trials each with a short rest break between the blocks. Congruent and incongruent trials were randomly and equally distributed. Only complete alternation sequences were employed to minimize both positive and negative priming confounds. That is, neither the ink nor the word color for the color-word Stroop task, and neither the direction nor the arrow position for the spatial Stroop task, used on the current trial were repeated in either way (ink or word, and direction or position) on the subsequent trial (see Puccioni and Vallesi, 2012a,b, for details).

Prior to the experimental blocks, participants completed 16 training trials. They had to perform correctly on at least 10 out of the 16 trials to proceed to the subsequent experimental blocks. Otherwise, the practice had to be repeated until such a criterion was reached.

### Data Analysis

Since the distributions of the response times (RTs) and accuracy scores were skewed and/or kurtotic, we respectively applied logarithmic and arcsine square root transformations to improve normality and reduce skewness. The use of log-transformed RTs also enabled controlling for possible unspecific effects of generalized slowing (e.g., Ben-David et al., 2014).

For the RT analysis, the first trial in each block (1.56% of all the trials) was discarded, as well as errors (5.70% of the remaining trials) and anticipations (RTs < 150 ms, <0.01% of the remaining trials). Additionally, trials following an error (5.26% of the remaining trials) were excluded to avoid post-error slowing confounds (Burns, 1965). Finally, for each participant, trials with an RT above or below 2 *SD* from their individual task mean condition were treated as outliers and discarded from the RT analysis (4.61% of the remaining trials). For the accuracy analysis, the first trial in each block was removed.

For both correct RTs and accuracy scores, we computed verbal and spatial Stroop effects by calculating the difference between congruent and incongruent trials and then assessed their statistical significance by means of one-sample *t* test against zero. The Cohen’s *d* was used as a measure of the effect size (Cohen, 1977). We also tested the reliability of our verbal and spatial (RT and accuracy) Stroop effects by computing split-half correlations corrected with the Spearman–Brown formula. This procedure is critical when performing correlation/regression analyses, since low observed correlations might result from poor reliabilities of the used measures and not from the lack of a true relationship between variables. To this aim, for both verbal and spatial tasks, we randomly divided congruent and incongruent trials of each participant into two subgroups of equal size; we then computed the spatial and verbal Stroop effects for each half as described above and calculated the corresponding Spearman–Brown-corrected reliability indexes. All reliability indexes were obtained from 5000 different randomizations of the trials.

Next, the following analyses were performed. First, to investigate the linear relationship between direction of handedness and Stroop performance, we ran non-parametric analyses (i.e., based on rank-transformed data) since the distribution of the EHI scores was negatively skewed and not normally distributed (skewness = −1.55; kurtosis = 1.02; Shapiro-Wilk’s *W* = 0.717, *p* < 0.001). Specifically, we performed a non-parametric linear regression analysis between the participants’ rank-transformed EHI scores and their rank-transformed verbal and spatial Stroop effects. Note that the resulting regression parameter is equivalent to the non-parametric Spearman’s correlation coefficient *ρ*. Moreover, to assess whether the relationship between direction of handedness and Stroop performance was modulated by the cognitive domain, we carried out non-parametric general linear model (GLM) analyses with the participants’ rank-transformed verbal and spatial Stroop effects as dependent variables, Domain as a within-participants factor, and the rank-transformed EHI scores as a continuous predictor.

Second, to compare the effects of both consistency and direction of handedness in the same analysis, we carried out additional non-parametric regression analyses by including two regressors for the linear and the quadratic effect of the participants’ EHI scores in explaining their variability in the spatial and verbal Stroop tasks. For each Stroop effect, the fit of this quadratic model to the data was compared to that of the simpler linear model by means of an *F* test (Δ*F*) for *R*^2^ change. In this way, we were able to assess whether the inclusion of the quadratic term was justified and, hence, to test the specific contribution of consistency of handedness over and above that of direction. Indeed, if consistency matters, the quadratic model should show a better fit to the data as compared to the linear one, with a U-shaped relationship between EHI scores and Stroop effects. Specifically, a U-shaped finding would indicate a difference between Stroop performance of inconsistent handers with respect to that of both consistent left- and consistent right-handers.

## Results

### Response Times

The RT analysis confirmed the traditional Stroop task results with reliable verbal (*M* = 0.057, *SD* = 0.039, corresponding to a mean untransformed raw effect of 92.89 ms, *SD* = 70.64 ms; *t*_(286)_ = 24.75, *p* < 10^−72^, *d* = 1.46) and spatial Stroop effects (*M* = 0.084, *SD* = 0.028, corresponding to a mean untransformed raw effect of 109.40 ms, *SD* = 53.50 ms; *t*_(286)_ = 50.11, *p* < 10^−142^; *d* = 2.96). The correlation between the two Stroop effects was not significant (*ρ* = −0.008, *t*_(285)_ = −0.14, *p* = 0.888), a result that suggests some degree of independence in the cognitive processes underlying verbal and spatial Stroop tasks. The spatial and verbal RT Stroop effects had a good split-half reliability (respectively, median = 0.767 and 0.738, two-sided 95% confidence interval = [0.709 0.810] and [0.673 0.785]), making their use in the subsequent regression analysis appropriate. It is important to note that the global EHI score has also been shown to have good test-retest reliability (e.g., Ransil and Schachter, 1994).

The non-parametric regression analyses between the verbal and spatial Stroop effects and the EHI scores showed an opposite pattern of results: there was a negative correlation for the verbal Stroop task (*ρ* = −0.174, *p* = 0.003) and a positive correlation for the spatial Stroop one (*ρ* = 0.121, *p* = 0.041). These results indicate that in the verbal domain the Stroop effect was reduced for participants with more positive EHI scores, whereas in the spatial domain the Stroop effect was reduced for participants with less positive (or negative) scores. Moreover, the GLM analysis revealed a significant interaction between cognitive domain and direction of handedness (*F*_(1,285)_ = 12.78, *p* < 0.001, $\eta_{\text{p}}^{2}$ = 0.04), confirming that the verbal and spatial Stroop effects were predicted by the EHI scores in an opposite way. To further verify that the correlations between the Stroop effects and the EHI scores in the verbal and spatial domains were significantly different, we also used the statistical test for comparing overlapping correlations from dependent groups described in Diedenhofen and Musch (2015). By overlapping correlations, it is meant that the same variable (in our case the EHI scores) is common to both correlations. Such a test confirmed that the two correlations observed here were statistically different (Meng et al’s., 1992; *Z* = −3.434, *p* < 0.001).

We then fitted the participants’ verbal and spatial Stroop effects with two non-parametric models including two regressors for the linear and the quadratic effect of the participants’ EHI scores. This was done in order to evaluate whether these quadratic models explained participants’ Stroop performance better than the simpler linear ones. The improvement of the model fit due to the inclusion of the quadratic term was not significant for the verbal Stroop effect (Δ*F*_(1,284)_ = 1.95, corrected *p* = 0.327) and only marginally significant for the spatial one (Δ*F*_(1,284)_ = 4.57, corrected *p* = 0.066), but the increase in *R*^2^ was negligible in both cases (respectively, <0.01 and 0.02). This analysis, thus, showed that the quadratic model accounting for the effects of both consistency and direction of handedness together did not provide a more adequate explanation of the participants’ verbal Stroop effect as compared to the simpler linear model describing the effect of direction of handedness alone. There was, instead, a marginal significant improvement of the model for the inclusion of the quadratic term in the case of the spatial Stroop task.

**Figure 1:**
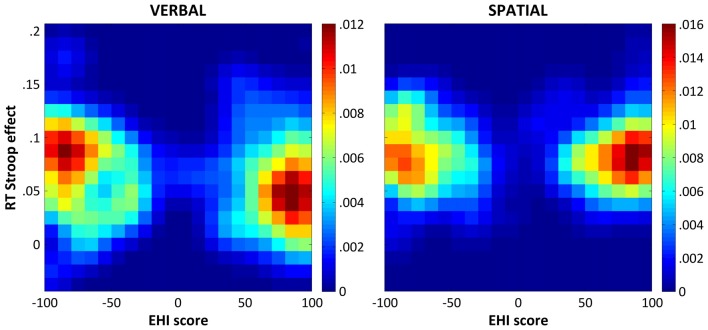
Bivariate distributions of the response time (RT) Stroop effects and the Edinburgh Handedness Inventory (EHI) scores. The figure shows the bivariate distributions of the participants’ verbal (left panel) and spatial (right panel) Stroop effects (*y* axis) as a function of their EHI scores (*x* axis) as derived from 10,000 random subsets matching the number and the EHI scores of participants with negative and positive EHI scores in our sample (see “Results” section for details). The bivariate distributions were smoothed with a Gaussian kernel with a full-width half-maximum of 2 × 2 bins, which corresponds to a Stroop effect of 0.025 and an EHI score of 10. The colorbars indicate probability densities.

Finally, to control for a possible rightward bias that might have influenced the correlation results, we performed an additional analysis in which the proportion of positive and negative EHI scores was exactly matched. That is, for each participant with a negative EHI score, one participant with the opposite EHI score was randomly selected. Since negative and positive values that could not be paired up were discarded, this procedure resulted in a total of 41 matched participant pairs. We then computed a Spearman correlation on this new dataset and repeated the same procedure on 10,000 random subsets. The two-sided 95% confidence interval (CI_95%_), which corresponds to an alpha level of 0.05, was finally computed. The results of this series of correlations remained the same as those described above for the color-word Stroop task (median *ρ* = −0.242, CI_95%_ = [−0.374 −0.107]), but not for the spatial Stroop task (median *ρ* = −0.021, CI_95%_ = [−0.159 0.114]). For illustrative purpose, Figure 1 shows the bivariate distributions of the participants’ verbal and spatial Stroop effects as a function of their EHI scores resulting from the 10,000 matched random subsets derived as described above.

### Accuracy

The analysis conducted on the accuracy scores paralleled the RT findings in that there were significant verbal (*M* = 0.081, *SD* = 0.115, corresponding to a mean untransformed raw effect of 3.88%, *SD* = 6.21%; *t*_(286)_ = 11.86, *p* < 10^−25^, *d* = 0.70) and spatial (*M* = 0.202, *SD* = 0.125, corresponding to a mean untransformed raw effect of 6.99%, *SD* = 7.16%; *t*_(286)_ = 27.33, *p* < 10^−80^, *d* = 1.61) Stroop effects also in the accuracy data. Differently from what observed for the RT data, however, the correlation between the two accuracy Stroop effects was significant (*ρ* = 0.182, *t*_(285)_ = 3.13, *p* = 0.002). The spatial and verbal accuracy Stroop effects had good reliability indexes (respectively, median = 0.759 and 0.529, CI_95%_ = [0.704 0.804] and [0.417 0.620]), albeit the verbal one was slightly lower than that found for the corresponding RT Stroop effect.

The non-parametric regression analyses between the verbal and spatial Stroop effects and the EHI scores showed a pattern of results that differed from that observed for the RT Stroop effects in the following aspect. While there was a significant negative correlation for the verbal Stroop task (*ρ* = −0.224, *p* < 0.001), the correlation for the spatial Stroop one failed to reach significance (*ρ* = −0.035, *p* = 0.560). However, the GLM analysis confirmed a significant interaction between cognitive domain and direction of handedness (*F*_(1,285)_ = 6.38, *p* = 0.012, $\eta_{\text{p}}^{2}$ = 0.02), showing that the verbal and spatial accuracy Stroop effects were predicted by the EHI scores in a significantly different way. Moreover, the test for comparing overlapping correlations confirmed that the correlation for the verbal task was different than that for the spatial one (Meng et al’s., 1992; *Z* = −2.522, *p* = 0.012).

Paralleling the results on the RT Stroop effects, the improvement of the model fit due to the inclusion of the quadratic term was not significant for the verbal and spatial Stroop effects (both Δ*F*s_(1,284)_ < 2.82, *p*s > 0.583), with negligible *R*^2^ increase in both cases (both < 0.01).

**Figure 2:**
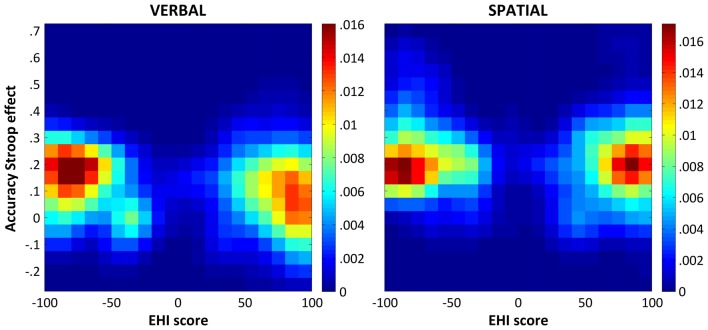
Bivariate distributions of the accuracy Stroop effects and the EHI scores. The figure shows the bivariate distributions of the participants’ verbal (left panel) and spatial (right panel) accuracy Stroop effects (*y* axis) as a function of their EHI scores (*x* axis) as derived from 10,000 random subsets matching the number and EHI scores of participants with negative and positive EHI scores in our sample (see “Results” section for details). The bivariate distributions were smoothed with a Gaussian kernel with a full-width half-maximum of 2 × 2 bins, which corresponds to a Stroop effect of 0.1 and an EHI score of 10. The colorbars indicate probability densities.

As for the RT Stroop effects, we performed an additional analysis in which the proportion of participants with positive and negative EHI scores was exactly matched in 10,000 random subsets of the data. The results of this series of correlations confirmed those described above. Indeed, the correlation for the color-word Stroop task was significant (median *ρ* = −0.281, CI_95%_ = [−0.406 −0.143]), whereas that for the spatial Stroop task was not (median *ρ* = −0.083, CI_95%_ = [−0.235 0.069]). For illustrative purpose, Figure 2 shows the bivariate distributions of the participants’ verbal and spatial accuracy Stroop effects as a function of their EHI scores resulting from 10,000 matched random subsets.

## Discussion

The goal of the present study was to explore the relationship between handedness and cognitive functioning. Our working hypothesis derives from recent work showing that non-right-handers should perform comparatively better on those tasks requiring greater inter-hemispheric collaboration and access to the right hemisphere, such as the spatial version of the Stroop task (Prichard et al., 2013). They should instead perform worse on tasks for which inter-hemispheric interaction is not advantageous to performance and that require preferential access to the left hemisphere, such as the verbal version of the Stroop task (Christman, 2001). If this were true, handedness research could also shed some light on current accounts of executive functions, according to which these functions may be differently lateralized in the brain (e.g., Stuss, 2011; Vallesi, 2012), with mechanisms underlying the Stroop task assumed to mostly engage the left hemisphere (e.g., Floden et al., 2011; Gläscher et al., 2012; Geddes et al., 2014; Cipolotti et al., 2016).

The main findings of this study can be summarized as follows. In line with our predictions, we found that handedness modulated performance on verbal and spatial Stroop tasks in opposite ways. Indeed, the regression analyses showed that right-handers were better able to reduce Stroop interference in the verbal task compared to non-right-handers, in terms of both RT and accuracy, whereas non-right-handers exhibited an advantage in the spatial Stroop task, albeit for RT only. In other words, the relationship between handedness and verbal-spatial Stroop performance was accounted for by linear relationships with opposite signs, thus showing that direction of handedness played a critical, but differential, role in the two Stroop tasks. This finding was further corroborated by follow-up non-parametric GLM analyses, which confirmed that the verbal and spatial Stroop effects were predicted by the participants’ EHI scores in a significantly reversed manner, for both RT and accuracy.

In order to disentangle direction and consistency of handedness, we also fitted the participants’ verbal and spatial Stroop effects with both linear and quadratic models. This allowed clarifying that, especially for the verbal domain, consistency of handedness did not account for the data better than direction alone. Contrarily, we should have found a different pattern for inconsistent/mixed-handers compared to either consistent left or consistent right-handers. Moreover, we tested whether the relatively small number of left-handed participants (*N* = 52) in our sample influenced the results obtained in the two Stroop tasks. To control for this possible bias, we equated the number of participants with negative and positive EHI scores and performed correlations on these new datasets, repeating such a procedure 10,000 times. This analysis refined the following points. For the color-word Stroop task, and hence in the context of the verbal domain only, the more and the stronger one is right-lateralized, the greater the ability to resist interference from competing word reading information. Such an advantage was present for both RT and accuracy data. It can be then concluded that the significant relation between handedness and Stroop performance observed in the verbal domain was reliable and robust in spite of the common rightward bias in the original distribution of the EHI scores. By contrast, in the spatial domain, the significant correlation result we found for the RT data was not confirmed by the accuracy analysis. Also, the RT advantage related to handedness disappeared in the correlation analysis of the spatial Stroop task when equating the number of participants with negative and positive EHI scores.

Two non-mutually exclusive explanations can account for the latter finding. The first one considers that the correlation we found in the spatial Stroop task could have been driven not only by consistent left-handers but, at least partly, also by inconsistent/mixed-handers (i.e., those participants with EHI scores in the middle of the distribution), who were under-represented in the control analysis matching the number of participants with positive and negative EHI scores (see Figures 1, 2). Lending support to this hypothesis, the inclusion of the quadratic model in the regression analysis was not significant for the verbal domain, while it was marginally significant for the spatial one suggesting that consistency of handedness could contribute to explain Stroop performance in the spatial domain. The second consideration is that, relative to the verbal domain, the relation between handedness and Stroop performance in the spatial one was more prone to be biased by the rightward asymmetry in the distribution of the EHI scores and the relatively low number of left-handers. One might therefore speculate that what we observed in the spatial Stroop task might simply reflect a statistical artifact and not a real advantage related to direction of handedness. Enrolling a higher number of left-handers to control for the rightward bias in the distribution of the EHI scores in future work is, thus, necessary to assess the impact of both direction and consistency of handedness on spatial Stroop performance. In any case, it is important to underscore here that our main finding was that handedness significantly modulated performance on verbal and spatial Stroop tasks in relatively opposite ways, as shown by the GLM analysis and the Meng’s test, a pattern of results that supports the idea that hand preference exerted a differential influence on the two types of tasks.

An alternative explanation for the differences observed between spatial and verbal Stroop performance as a function of handedness is related to task difficulty. That is, it could be argued that since the spatial Stroop task was more difficult than the verbal Stroop task (in terms of higher Stroop effect), non-right-handers outperformed right-handers when overall task difficulty was relatively high, while the opposite was true when overall task difficulty was low. This would fit well with previous studies showing that hemispheric interactions are beneficial for relatively difficult tasks, while within-hemisphere processing is advantageous for relatively simple tasks (e.g., Banich and Belger, 1990; Weissman and Banich, 1999, 2000). Despite its apparent plausibility, however, this explanation cannot apply to our data to the extent that, in terms of overall task difficulty, the verbal Stroop task was indeed relatively more difficult than the spatial one^2^. Accordingly, general task difficulty does not offer a valid framework to explain our findings. It should also be noted that our findings cannot be simply attributed to low-level verbal or spatial abilities, as these abilities were not correlated to the EHI scores^3^. The results reported here were specific to the Stroop effect, as also suggested by the fact that when congruent and incongruent conditions were taken separately, no significant correlations with the EHI scores were observed for RT data, while only one significant correlation emerged for accuracy^4^. In particular, these control analyses showed that non-right-handers had lower accuracy in the incongruent condition of the color-word Stroop task only, a result that is still in line with our hypothesis of worse performance for non-right-handers on the verbal condition, for which inter-hemispheric interaction was assumed not to be useful for performance. Thus, this result does not affect our main conclusions.

Although our hypothesis on the common involvement of the left hemisphere for both Stroop tasks could seem somewhat speculative, there is evidence that bolsters it. In a recent resting-state electroencephalographic (EEG) study, Ambrosini and Vallesi (2017) used the same color-word and spatial Stroop tasks as the ones reported here. They found that participants with stronger resting-state-related activity in left-lateralized prefrontal regions were more able to resolve Stroop interference in both verbal and spatial tasks, which were administered at a later time with respect to the EEG session. Left-lateralized activations in the spatial Stroop task were also reported by Zoccatelli et al. (2010) in their fMRI study.

Along the same line, in a previous fMRI study, we investigated another executive function relying on control processes, namely, task-switching ability and, like here, spatial and verbal tasks were administered to the same participants (Vallesi et al., 2015). Our results showed a left-lateralized involvement of fronto-parietal regions for the verbal task and a more bilateral pattern for the spatial task. Importantly, a conjunction analysis revealed that, together with the bilateral supplementary motor area, task-switching in both spatial and verbal tasks activated left fronto-parietal regions. It thus seems likely that the left hemisphere is specialized for those cognitive control processes underlying resistance to interference and cognitive flexibility (e.g., Derrfuss et al., 2005; Ambrosini and Vallesi, 2016), but that it may interact with the right hemisphere as a function of the (spatial) nature of the task to be performed (see also Babcock and Vallesi, 2015). Future studies should employ other types of conflicting stimuli differently lateralized to the two hemispheres to further test our predictions and check their generalizability. Moreover, it is highly recommended to complement these behavioral observations with neural data to gain more direct insights into the brain asymmetries underlying handedness and cognitive control task-performance within the same individuals.

In sum, the behavioral dissociations reported here confirmed our starting hypotheses. Indeed, replicating previous findings (Christman, 2001; but see Beratis et al., 2010), we found that non-right-handers showed significantly greater interference when faced with the verbal Stroop task for which inter-hemispheric interaction was not useful and interference had to be resolved mainly by their left side of the brain. Conversely, they performed relatively better, at least in terms of RT, when confronted with the spatial Stroop task, for which access to right hemisphere processes was needed and greater collaboration between the two hemispheres was beneficial to performance.

To conclude, the current study suggests that handedness may be a useful tool to also test predictions derived by neural models that fractionate high-level cognitive processes along the left-right axis of the human brain. More importantly, it provides evidence in favor of a growing literature arguing that handedness may help explain individual differences in cognitive performance.

## Author Contributions

MC drafted the manuscript and was involved in all subsequent revisions. She was also involved in data collection and data analysis. EA performed statistical analysis, drafted the manuscript and provided additional revisions to the manuscript. AV was involved in the conception of the work and provided ongoing contributions and feedback throughout the experimental process. He also provided additional revisions to the manuscript. All the authors have approved the final version of the manuscript and agree to be accountable for all aspects of the work.

## Conflict of Interest Statement

The authors declare that the research was conducted in the absence of any commercial or financial relationships that could be construed as a potential conflict of interest.

## Footnotes

^1^One might argue that, despite these extra participants were not aware of the purpose of our study, their recruitment procedure could have introduced some biases such as a stronger motivation of non-right-handers compared to right-handers in performing the task. However, we believe this was not the case here because a specifically higher motivation in non-right-handers, if present, should have equally influenced verbal and spatial Stroop tasks, and therefore cannot explain the differences we found between the two domains as a function of handedness.

^2^The RTs for the congruent and, importantly, the incongruent conditions of the spatial Stroop task were both significantly lower than those in the congruent condition of the verbal Stroop task (respectively, *t*_(286)_ = 24.46 and 6.89, *p* < 10^−71^ and 10^−10^, *d* > 1.44 and 0.40), suggesting that the spatial Stroop task was actually easier than the verbal one.

^3^Additional control non-parametric regressions failed to find significant correlations between EHI scores and either RT or accuracy measures of both low-level verbal (respectively, *ρ* = −0.054 and 0.101, *p* = 0.361 and 0.087) and spatial (respectively, *ρ* = 0.096 and 0.035, *p* = 0.103 and 0.553) abilities, which were computed, respectively, as the mean RT or accuracy collapsed over congruent and incongruent conditions of the verbal and spatial Stroop tasks.

^4^The *ρ* correlations between EHI scores and RT data were as follows: spatial congruent = 0.077, *p* = 0.193; spatial incongruent = 0.106, *p* = 0.071; verbal congruent = −0.025, *p* = 0.678; verbal incongruent = −0.088, *p* = 0.136. The *ρ* correlations between EHI scores and accuracy were as follows: spatial congruent = −0.023, *p* = 0.690; spatial incongruent = 0.051, *p* = 0.388; verbal congruent = −0.038, *p* = 0.520; verbal incongruent = 0.187, *p* = 0.001.
